# Phase I/II study of intraperitoneal docetaxel plus S-1 for the gastric cancer patients with peritoneal carcinomatosis

**DOI:** 10.1007/s00280-013-2122-0

**Published:** 2013-02-20

**Authors:** S. Fushida, J. Kinoshita, M. Kaji, Y. Hirono, F. Goda, Y. Yagi, K. Oyama, Y. Sudo, Y. Watanabe, T. Fujimura, Society for the Study of Peritoneal Carcinomatosis in Gastric Cancer

**Affiliations:** 1 Department of Gastroenterological Surgery, Kanazawa University Hospital, Takara-machi Kanazawa, 920-8641 Japan; 2 Department of Surgery, Toyama Prefecture Central Hospital, Nishinagae Toyama, 930-0975 Japan; 3 Department of Gastroenterological Surgery, Fukui University Hospital, Matsuoka Yoshida, 910-1193 Japan; 4 Division of Cancer Center, Kagawa University Hospital, Miki-machi Kida, 761-0793 Japan; 5 Department of Surgery, Higashiohmiya General Hospital, Higashiohmiya Saitama, 337-0051 Japan; 6 Department of Surgery, Ehime University Hospital, Shitsukawa Toon, 791-0295 Japan

**Keywords:** Intraperitoneal chemotherapy, Docetaxel, S-1, Gastric cancer, Peritoneal carcinomatosis

## Abstract

**Purpose:**

We designed a phase I/II trial of intraperitoneal (IP) docetaxel plus S-1 to determine the maximum tolerated dose (MTD) and recommended dose (RD) and to evaluate its efficacy and safety in gastric cancer patients with peritoneal carcinomatosis (PC).

**Methods:**

Patients with PC confirmed by laparoscopy or laparotomy received IP docetaxel on days 1 and 15 and S-1 (80 mg/m^2^) on days 1–14 every 4 weeks.

**Results:**

In the phase I part (*n* = 12), each cohort received escalating doses of docetaxel (35–50 mg/m^2^); the MTD was determined to be 50 mg/m^2^ and the RD was determined to be 45 mg/m^2^. Dose-limiting toxicities included grade 3 febrile neutropenia and grade 3 diarrhea. In the phase II part (*n* = 27), the median number of courses was 4 (range 2–11). The 1-year overall survival (OS) rate was 70 % (95 % confidence interval 53–87 %). The overall response rate was 22 % and peritoneal cytology turned negative in 18 of 22 (81 %) patients. The most frequent grade 3/4 toxicities included anorexia (19 %), neutropenia (7 %), and leukopenia (7 %).

**Conclusion:**

IP docetaxel plus S-1 is active and safety in gastric cancer patients with PC.

## Introduction

Gastric cancer is one of the major causes of cancer death worldwide; however, recent advances in systemic chemotherapy regimens using combinations of novel anti-neoplastic agents have shown encouraging tumor response rates and survival for patients with unresectable or metastatic gastric cancer.

In patients with positive peritoneal cytology and no macroscopic peritoneal tumors, radical surgery followed by postoperative S-1 showed good results with a median survival time (MST) of 705 days and 2-year survival rate of 47 % [1]. But the prognosis of patients with macroscopic peritoneal carcinomatosis (PC), which is responsible for about 60 % of all the deaths from gastric cancer [2, 3], is extremely poor with MST of 3–6 months [4, 5].

As the reason for this, only limited amounts of drugs reach the peritoneal cavity after intravenous administration due to the peritoneal-blood barrier [6]. So far, aggressive methods have been tried to treat PC, such as cytoreductive surgery (CRS) plus hyperthermic intraperitoneal chemotherapy (HIPEC) [7]. A recent phase III study showed that patients treated with CRS plus HIPEC had superior survival to those treated with CRS alone, and the MST for CRS plus HIPEC was 11.0 months [8]. However, because of the high morbidity and mortality rates, these aggressive treatments should only be used for highly selected patients. Thus, neither regimen has been accepted as the standard chemotherapy for PC, and a new-multidisciplinary approach for gastric cancer with PC is needed.

The oral anticancer drug S-1 is a fluoropyrimidine derivative, combining tegafur with two modulators [9]. In recent phase III studies, S-1 showed response rates of 27–31 % and MST of 10.5–11.4 months [10, 11], and it is considered to be a pivotal agent for gastric cancer in Japan. S-1 was also highly effective against gastric PC due to the higher concentrations of 5-FU and CDHP achieved in peritoneal tumors than in plasma [12].

Docetaxel, which binds to tubulin, leading to microtubule stabilization, and mitotic arrest [13], has been widely used in the treatment of gastric cancer with response rates of 16–24 % when used as a single agent in phase II trials [14, 15]. Furthermore, docetaxel has high sensitivity against diffuse-type adenocarcinoma, which is a common type of peritoneal tumor, and some of these compounds, when administered intravenously, are transported into the peritoneal cavity [16, 17]. These findings suggest that combination therapy using S-1 and intravenous docetaxel is also one of the candidates for first-line treatment for PC.

Intraperitoneal administration of docetaxel (IP docetaxel) was developed to enhance antitumor activity against PC by maintaining a high concentration of the drug. Although we previously reported the efficacy and safety of weekly IP docetaxel monotherapy [18], there have been few clinical trials using IP docetaxel plus S-1 with accurate estimation of peritoneal disease. In this study, we conducted a phase I/II study of IP docetaxel plus S-1 to develop a safe and effective treatment for gastric cancer patients with PC.

## Patients and methods

### Eligibility

The patients enrolled in this study had histologically confirmed with PC. Before enrollment in the study, PC was confirmed by either laparoscopy or laparotomy. PC was classified according to the criteria of the Japanese Research Society for Gastric Cancer [19] as follows: P1, cancerous implants to the region directly adjacent to the stomach peritoneum (cranial to the transverse colon) including the great omentum; P2, several scattered metastases to the distant peritoneum and ovarian metastasis alone; and P3, numerous metastases to the distant peritoneum. Photographs of peritoneal lesions were taken before and after treatment to estimate an objective response. Other criteria for inclusion were: (1) Eastern Cooperative Oncology Group (ECOG) performance status of 0–2; (2) age ranged between 20 and 75 years old; (3) adequate bone marrow, liver, and renal functions as defined by WBC >4,000, <12,000/mm^3^, PLT >100,000/mm^3^, Hb >8.0 g/dl, AST/ALT <2 times institutional upper limit, total bilirubin <1.5 mg/dl, creatinine <1.5 mg/dl and creatinine clearance >60 mL/min; (4) no significant cardiac disease evident on electrocardiogram; and (5) expected survival period >3 months.

The exclusion criteria were the following: (1) previous treatment for gastric cancer; (2) coexistence of another malignant neoplasm; (3) a history of reactions to drugs; (4) massive ascites and/or pleural effusion; and (5) brain metastasis.

This study was approved by the Institutional Review Boards of each institution, and the procedures were performed in accordance with the Declaration of Helsinki. All patients provided written informed consent to participation in the study in accordance with the institutional guidelines.

### Treatment schedule

An initial laparoscopy or mini-laparotomy was performed under general anesthesia in patients with advanced gastric cancer diagnosed histologically or patients with peritoneal recurrence diagnosed by imaging.

Palliative surgery for tumor reduction was not carried out. A peritoneal access port was implanted in the subcutaneous space of the lower abdomen, with a catheter placed in the pelvic cavity.

S-1 was administered orally at a fixed dose of 40 mg/m^2^ twice daily on days 1–14 every 4 weeks. Docetaxel was administered with 1,000 ml of 0.9 % sodium chloride solution via the implanted peritoneal access port for 2 h after standard premedication on days 1 and 15. Each cycle was performed every 4 weeks.

Clinical trials of IP docetaxel as a single agent revealed the RD of 45 mg/m^2^ weekly on days 1, 8, and 15 during the 4-week cycle for advanced gastric cancer patients [18]. In the present study, we planned combination chemotherapy using IP docetaxel plus S-1, which was thought to be more toxic than IP docetaxel monotherapy. Therefore, during phase I, the initial dose of docetaxel was 35 mg/m^2^ (Level 1) and the dose was escalated by 5 mg/m^2^ for each dose level up to 50 mg/m^2^ (Level 4).

The DLTs were defined as follows: (1) grade 4 hematological toxicity, (2) transfusion of platelets for thrombocytopenia, (3) grade 3 neutropenia with infection or fever >38.0 °C, (4) grade 3 or greater non-hematological toxicity with the exception of loss of appetite, nausea, and vomiting, and (5) treatment delay of more than 2 weeks following the last administration of docetaxel.

At least three patients were to be started at dose level 1: (1) the dose was defined as the maximum tolerated dose (MTD) when all patients developed DLT; (2) when one or two of three patients developed DLT, three other patients were enrolled, (3) when more than three of six patients developed DLT, the dose was defined as MTD; (4) when fewer than two of six patients developed DLT, the dose was increased to the next level. Assessment of DLT was conducted during the first two treatment cycles.

Phase II was performed using the RD determined during phase I. The treatment course was repeated until observation of unacceptable toxicity or disease progression.

After 2 treatment cycles, either second laparoscopy or laparotomy was scheduled to evaluate the effect of the treatment on PC. Surgical resection was performed for macroscopically curative operation according to second laparoscopic finding. Treatment after disease progression or surgery was at the physician’s discretion.

### Evaluation of tumor response

Peritoneal carcinomatosis is considered to be a non-evaluable lesion because it is difficult to detect PC by conventional radiological examinations. In this study, we developed new response criteria for treatment against PC: Complete response (CR), no detection of cancer cells in the peritoneal cytology and disappearance of all peritoneal tumors macroscopically and histologically; Partial response (PR), no detection of cancer cells in the peritoneum cytology and at least a 50 % decrease in the sum of the longest diameter of a peritoneal tumor using photographs of peritoneal lesions taken to confirm an objective response before and after treatment; Stable disease (SD), an insufficient decrease in the sum of the longest diameter of peritoneal tumor using photographs of the peritoneal lesions; Progressive disease (PD), exacerbation of a peritoneal tumor or the appearance of new peritoneal tumor lesions.

Peritoneal tumor response was evaluated using intraperitoneal photographs which were taken in the first and second laparoscopy according to the aforesaid new response criteria by the physician’s discretion. If peritoneal deposits were found, biopsy was performed to distinguish fibrosis from metastatic nodule.

Measurable lesions of the tumor response were evaluated after 2 treatment cycles using CT scans according to the Response Evaluation Criteria in Solid Tumors (RECIST) ver. 1.0.

### Phase II study: statistical planning and analysis

The primary endpoint for the phase II part of the study was the 1-year overall survival (OS) rate, and the secondary endpoints were the overall response rate (ORR), efficacy against malignant ascites, and safety.

The required number of patients was calculated according to the Southwest Oncology Group One Arm Survival program [20]. Recent studies in advanced or metastatic gastric cancer including patients with PC showed a 1-year OS rate of 50 % [10, 11, 21]. A 1-year OS rate of 70 % could be expected, as 5 of 7 patients survived >1 year in our pilot study with this regimen. Assuming a null hypothesis of 50 % and an alternative hypothesis of 70 % with one-sided type I error of 0.05 and power of 0.8, with an accrual time of 2 years and follow-up of 1 year after closure of recruitment, it was necessary to enroll 23 fully assessable patients. The 1-year survival rate was calculated using the Kaplan–Meier method. Adverse events were graded according to the National Cancer Institute-Common Terminology criteria for Adverse Events version 3.0.

## Results

### Phase I

#### Patients

Twelve patients were enrolled in the phase I study between February 2007 and October 2007 (Table 1). All patients received at least 2 courses of therapy. ECOG performance status was 0–1 for all patients. P1 was observed in one patient, P2 in 6 patients, and P3 in five patients. Two patients had undergone prior gastrectomy and had also experienced peritoneal recurrence. Ten patients retained the primary tumor. One patient had ovarian metastasis and one patient had liver metastasis. All patients underwent laparoscopy for diagnosis.

**Table 1:** Patients characteristics

Characteristics	Phase I	Phase II
No. of patients	12	27
Sex		
Male	10 (83 %)	14 (49 %)
Female	2 (17 %)	13 (51 %)
Median age in years (range)	63 (33–75)	66 (26–75)
ECOG performance status		
0	9 (75 %)	21 (78 %)
1	3 (25 %)	6 (22 %)
Prior treatment		
Gastrectomy	2 (17 %)	0 (0 %)
Chemotherapy	0 (0 %)	0 (0 %)
Metastatic organ		
Peritoneum	12 (100 %)	27 (100 %)
Peritoneal cytology	11 (92 %)	22 (81 %)
Lymph node	9 (75 %)	6 (22 %)
Liver	1 (8 %)	2 (7 %)
Lung	0 (0 %)	1 (4 %)
Ovary	1 (8 %)	0 (0 %)
PC grade		
P1	1 (8 %)	2 (7 %)
P2	6 (50 %)	7 (26 %)
P3	5 (42 %)	18 (67 %)

*ECOG* Eastern Cooperative Oncology Group, *PC* Peritoneal carcinomatosis

#### Toxicities

Chemotherapy toxicities per patient during the second cycle are summarized in Table 2. No patients showed toxicities of grade 4 or higher, while one patient enrolled at dose level 3 showed grade 3 neutropenia. At dose level 4, two experienced grade 3 febrile neutropenia, and one experienced grade 3 diarrhea. As all three patients treated at dose level 4 were deemed to have DLT, level 4 was considered as the MTD and level 3 (IP docetaxel 45 mg/m^2^) was defined as the RD for the ensuing phase II study.

**Table 2:** Toxicities at various dose levels of IP docetaxel plus S-1 during the first 2 cycles in the phase I part

Toxicity (CTCAE v. 3)	Dose of docetaxel							
	35 (*n* = 3)		40 (*n* = 3)		45 (*n* = 3)		50 (*n* = 3)	
	G1/2	G3/4	G1/2	G3/4	G1/2	G3/4	G1/2	G3/4
Anemia	2	0	2	0	2	0	0	0
Leukopenia	1	0	0	0	2	0	1	0
Neutropenia	1	0	0	0	1	1	2	0
Thrombocytopenia	0	0	0	0	0	0	0	0
FN	–	0	–	0	–	0	–	2
Nausea	2	0	1	0	1	0	3	0
Vomiting	0	0	0	0	0	0	0	0
Diarrhea	1	0	1	0	2	0	1	1
Abdominal pain	1	0	3	0	1	0	2	0

*FN* Febrile neutropenia

### Phase II

#### Patients

From November 2007 to October 2010, 27 patients were enrolled in the phase II study (Table 1): 14 (51.9 %) males and 13 (48.1 %) females with a median age of 66 years (range 26–75). All patients had an ECOG performance status of 0 or 1.

Metastatic sites included the peritoneum (100.0 %), lymph nodes (22.2 %), liver (7.4 %), and lung (3.7 %), and 22 patients (81.5 %) showed positive peritoneal cytology. None of the patients included in this study had undergone prior gastrectomy.

#### Efficacy

**Fig. 1:**
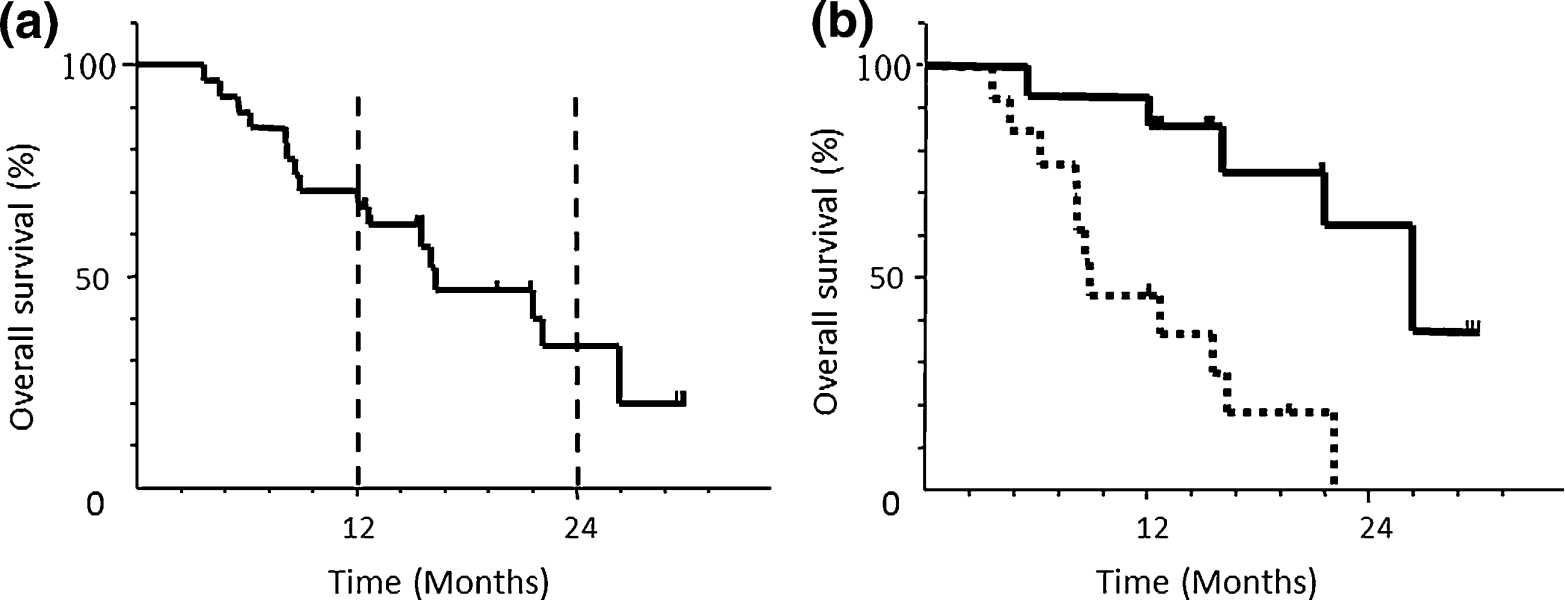
**a** Overall survival of all patients (*n* = 27) **b** Overall survival by peritoneal response. The *solid* and *dotted lines* present peritoneal responder (*n* = 14) and non-responder (*n* = 13)

A median of 4 cycles was administered with a range from 2 to 11. Combination chemotherapy was discontinued due to severe adverse events in two patients and due to disease progression in 11 patients. Figure 1a shows the overall survival time after the introduction of this combination therapy for all patients enrolled in the present study. The 1-year OS rate was 70.4 % (95 % CI 53.2–87.4 %), the 2-year OS rate was 33.4 % (95 % CI 11.8–55.0 %) at a median follow-up time of 27.6 months, and the MST was 16.2 months (95 % CI 8.4–22.1 months).

**Fig. 2:**
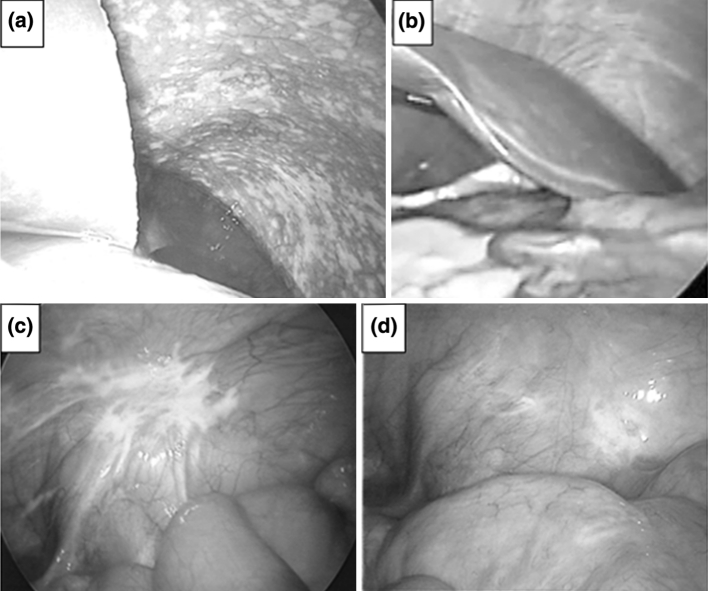
Examples of laparoscopic view *before* and *after* treatment. **a** Numerous peritoneal tumors on the diaphragm (*before* treatment), **b** No peritoneal tumor confirmed histologically (*after* treatment), **c** Peritoneal tumor on the *left lower quadrant* region (*before* treatment), **d** residual peritoneal tumor less than 50 % of diameter comparing the tumor in (**c**)

After 2 treatment cycles, all 27 patients were evaluated by second-look laparoscopy or laparotomy. Figure 2 shows a typical peritoneal tumor response. Before treatment, numerous metastases were observed on the diaphragm (panel a), and the peritoneal tumors disappeared macroscopically and histologically after treatment (panel b), which was assessed as CR. Panel d shows residual peritoneal tumors less than 50 % of the longest diameter compared to that before treatment (panel c), which was assessed as PR.

The peritoneal tumor response rate (RR) and overall RR were 51.9 % (CR3/PR11/SD10/PD3) and 22 % (CR0/PR6/SD2/PD3), respectively (Table 3). The 1-year and 2-year OS rates were 92.8 and 62.5 % for peritoneal responder group, and 46.1 and 0 % for non-responder group (Fig. 1b). Cancer cells ceased to be detected by peritoneal cytology in 18 of 22 (81.8 %) patients.

**Table 3:** Tumor response

Response	No. of patients	%
Overall response (*n* = 27)		
CR	0	0
PR	6	22
SD	2	7
PD	3	11
NE	16	60
Peritoneal tumor (*n* = 27)		
CR	3	11
PR	11	41
SD	10	37
PD	3	11
Peritoneal cytology (*n* = 22)		
Turned negative	18	82
Surgical resection (*n* = 14)		
R0	3	21
R1	11	79

Peritoneal tumor was assessed according to the new response criteria for the treatment against peritoneal carcinomatosis (see Table 1)

*NE* Not evaluated

#### Surgical treatment

According to the observations of the second-look laparoscopy, peritoneal responder (*n* = 14) underwent gastrectomy with lymph node dissection including removal of peritoneal deposit sites for macroscopic curative resection. Unfortunately, microscopic residual cancer cells in the tumor margin (R1 resection) were revealed in 11 of 14 patients. In this study, the tumor margin of residual peritoneal deposit site was defined positive, because it was impossible to remove it with more than 5 mm of sub-peritoneal tissue. Among the 14 patients who underwent surgery, postoperative complications occurred in 4 patients (29 %), anastomotic leakage in one patient, and pancreatic fistula in 3 patients. No surgery-related mortality (30 days mortality) was found.

#### Safety

All patients were assessable for toxicity. Table 4 summarizes chemotherapy toxicity per patient. The incidences of grade 3/4 hematological and non-hematological toxic effects were 7.4 and 18.5 %, respectively. The most frequent grade 3/4 toxic effects included neutropenia (7.4 %), leukopenia (7.4 %), and anorexia (18.5 %). None of the patients experienced grade 3/4 febrile neutropenia. Although abdominal pain related to IP infusion was observed in 5 patients (18.5 %), its severity was less than grade 2. Infection of the IP access port in one patient was the only complication related to the peritoneal access device. There was no chemotherapy-related mortality.

**Table 4:** Toxicity of chemotherapy in phase II (*n* = 27)

Toxicity (CTCAE v. 3)	Grade of adverse events				% of grade 3/4
	1	2	3	4	
Anemia	2	0	0	0	0
Leukopenia	1	2	1	1	7.4
Neutropenia	1	2	1	1	7.4
Thrombocytopenia	0	0	0	0	0
Nausea	3	0	0	0	0
Vomiting	0	0	0	0	0
Diarrhea	1	1	0	0	0
Anorexia	5	2	5	0	18.5
Stomatitis	0	1	0	0	0
Malaise	3	0	0	0	0
Alopecia	2	0	0	0	0
Abdominal pain	4	1	0	0	0

## Discussion

We have shown here that IP docetaxel plus S-1 is a highly active first-line chemotherapy regimen for advanced gastric cancer with PC.

In the phase I part of this study, we identified IP docetaxel at 45 mg/m^2^ on days 1 and 15 plus S-1 40 mg/m^2^ twice a day on day 1–14, every 28 days as the recommended treatment schedule for further clinical evaluation. This dose of IP docetaxel is the same dose as weekly IP docetaxel, which has already been described as monotherapy [18]. During dose escalation, DLT included 2 case of grade 3 febrile neutropenia and 1 case of grade 3 diarrhea during the 2nd cycle. In the present study, the assessment of DLT was conducted during the first two cycles, although the conventional phase I study evaluated toxicities in only the first cycle. This is the reason why patients should generally undergo a second staging laparoscopy after 2 cycles for the confirmation of the treatment effects on PC.

In the phase II part of this study, our combination regimen showed a 1-year OS rate of 70.4 % (95 % CI 53.2–87.4 %) with MST of 16.2 months (95 % CI 8.4–22.1 months). We obtained satisfactory results which considered the other phase II or III study for patients with unresectable or recurrent gastric cancer demonstrated 1-year OS rate around 50 %. Systemic chemotherapy based on fluorouracil for patients with peritoneal disseminated gastric cancer showed poorer 1-year OS rate between 20 and 40 %, respectively. Furthermore, cancer cells detected on peritoneal cytology disappeared in 18 of 22 (82 %) cases, and the second staging laparoscopy after 2 cycles of combined chemotherapy showed RR of 52 % and disease control rate (DCR) of 89 % according to the response criteria for the treatment of PC.

These superior results were due to the pharmacokinetic advantage of taxanes after regional delivery [18, 22–24]. Taxanes are absorbed through the openings of lymphatic system, such as the milky spots and the stomata which are important sites for the formation of peritoneal dissemination [25], due to their large molecular weight and fat solubility. Especially, IP paclitaxel showed a profound pharmacokinetic advantage 1,000 times higher than systemic administration.

The main problem of IPC is the limited depth of penetration of anticancer drugs directly into the tumor. Accordingly, optimum use of paclitaxel may consist of intraperitoneal and intravenous (IV) administration, because IP paclitaxel reaches the systemic circulation in only a small amount [26]. Actually, Ishigami et al. [27] established IP paclitaxel with S-1 plus IV paclitaxel as systemic chemotherapy, and the results were very encouraging similar to the present study.

In contrast, docetaxel, one of the taxanes, has a pharmacokinetic advantage after intraperitoneal delivery which is hundreds of times higher than systemic administration, and systemic AUC after IP is twice that after standard IV docetaxel [18]. These findings indicate that IP docetaxel has dual anticancer effects via the peritoneal surface and capillary blood supply. We also described that the mean value of the peak plasma concentration at 45 mg/m^2^ of IP docetaxel was extremely higher than the IC_50_ value of most gastric cancer cell lines [18, 28]. Therefore, in using IP docetaxel, it might be not necessary to perform intravenous administration as paclitaxel [26].

Fujiwara et al. [29] also reported the usefulness of IP docetaxel combined with S-1 for gastric cancer with PC. In a number of previous studies, the estimation of intraperitoneal information was unclear to disclose whether only cancer cell positive on peritoneal cytology or macroscopic peritoneal metastases exist before treatments. Thus, their results seem to reflect the high population of positive peritoneal cytology alone. In the present study, all patients underwent staging laparoscopy before chemotherapy, and to enhance the intraperitoneal efficacy, IP docetaxel was performed twice weekly, which was more frequent than in previous reports.

In our phase II part, peritoneal responder (*n* = 14) underwent surgical treatment. Although most of them were considered R1 resection, responder showed significant longer survival than non-responder (*p* = 0.005).

The most common adverse events in IV docetaxel combined with S-1 regimen were neutropenia and leucopenia, for which the incidences of grade 3/4 were 40–60 % [21, 30]. In the present study, only 2 patients (7.4 %) showed grade 3/4 neutropenia and leukopenia due to slow absorption of docetaxel from the peritoneal cavity in the systemic circulation. Grade 3 anorexia, however, occurred in a relatively high proportion of patients (18.5 %). This may be associated with severe PC in itself because nausea, which is closely correlated to anorexia, was observed in three patients with grade 1. As a unique toxic effect of IP docetaxel, abdominal pain was found in five patients who required no narcotic analgesia.

Although docetaxel and paclitaxel share major parts of their structures and mechanisms of action, there is only partial cross-resistance between these agents [31, 32]. Therefore, each IP taxanes (docetaxel, paclitaxel) may play a complementary role in case of treatment failure. It will be interesting to evaluate whether IP docetaxel really acts on patients who have failed IP paclitaxel.

In conclusion, our study suggested that IP docetaxel plus S-1 may be a novel treatment option for patients with PC in gastric cancer. More recently, a retrospective study of fluoropyrimidine (S-1 or capecitabine) plus cisplatin showed favorable results in patients with PC [33]. Further investigations, including controlled clinical trials comparing S-1 plus cisplatin and S-1 plus IP docetaxel used in the present study, are needed to confirm our findings.
